# Anatomical considerations in selective amygdalohippocampectomy techniques for refractory temporal lobe epilepsy: a cadaveric study with emphasis on white matter tract anatomy

**DOI:** 10.1007/s00276-024-03510-x

**Published:** 2024-11-27

**Authors:** Tomasz Andrzej Dziedzic, Michał Senger, Przemysław Kunert

**Affiliations:** Department of Neurosurgery, Medical University of Warsaw, Warsaw, Poland

**Keywords:** Amygdalohippocampectomy, Temporal epilepsy, Mesial temporal sclerosis, Subtemporal, Transsylvian, temporal lobectomy

## Abstract

**Purpose:**

Mesial temporal lobe epilepsy is a common form of focal drug resistant epilepsy in adults. Various mesial temporal lobe structures are integral in the genesis of temporal seizures and the hippocampal sclerosis is the primary neuropathological finding in these cases. Surgical treatment is considered the preferred management. This study aims to analyze the anatomical and surgical aspects of various resection techniques of selective amygdalohippocampectomy (SAHE)and clarify the critical anatomical landmarks and technical nuances associated which each method.

**Methods:**

Through dissection of five human head and brain specimens we evaluated three primary surgical approaches for SAHE—transsylvian, transcortical, and subtemporal — and additionally discussed laser interstitial thermal therapy (LITT). We examined the anatomical considerations of the temporal lobe and its white matter tracts, as well as the technical aspects of each approach.

**Results:**

The transcortical approach provides direct access to mesial structures but requires precise placement of the corticotomy based on hemisphere dominance to avoid arcuate fascicle and optic radiation. The subtemporal approach preserves all above white matter tracts but may risk interruption of the inferior longitudinal fasciculus. The transsylvian approach allows for comprehensive exposure but poses risks to tracts within limen insulae, namely uncinate and inferior fronto – occipital fascicles. Additionally, there is a risk to middle cerebral artery and its branches. LITT offers a minimally invasive alternative with comparable outcomes and reduced risk of cognitive side effects.

**Conclusion:**

Selective amygdalohippocampectomy and its variants, including LITT, are surgical strategies for managing mesial temporal lobe epilepsy. Each approach has distinct anatomical and technical considerations that influence the choice of a technique. Due to complex anatomy of temporal lobe and white matter tracts variability more research is essential for achieving favourable outcomes.

## Introduction

**Fig. 1:**
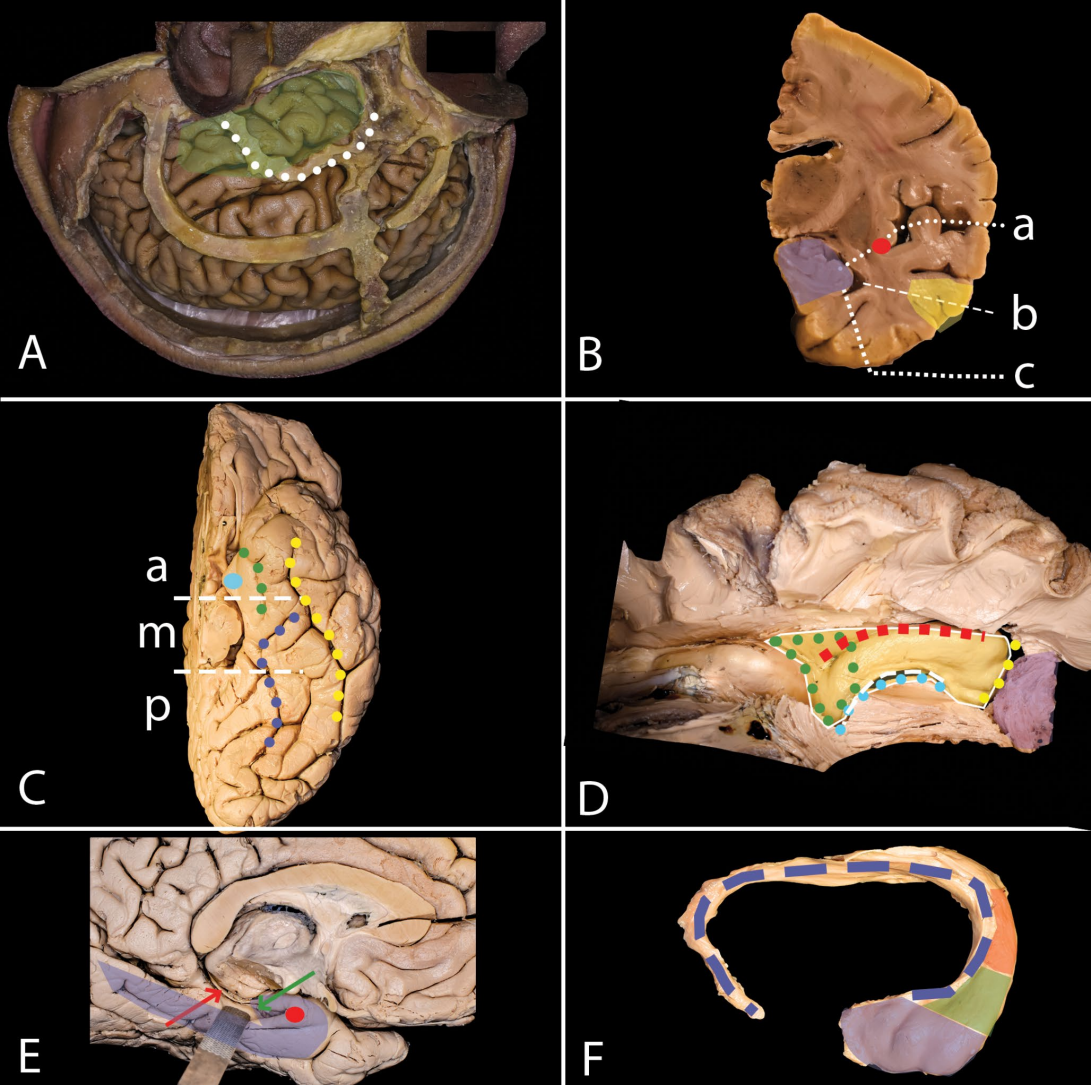
**A**-**F**. Overview of Temporal Lobe Anatomy. **A** The temporal lobe (green) is aligned with the squamous part of the temporal bone (white dots). **B** Mesial temporal structures (purple) lie beneath the middle temporal gyrus (yellow) and can be accessed via the Sylvian fissure (a - white dots), the inferior limiting sulcus (red dot), the middle temporal gyrus (b - white rectangle), or along the base of the temporal lobe (c - white squares). **C** The basal surface shows key landmarks: collateral sulcus (blue dots), uncus (light blue dot), rhinal sulcus (green dots), and lateral occipitotemporal sulcus (yellow dots), with anterior (a), medial (m), and posterior (p) segments. **D** The temporal horn (yellow) from above reveals structures like the atrium (green dots), collateral eminence (red squares), choroid fissure (light blue dots), fimbria of the fornix (white rectangle), the amygdala (purple), and the uncal recess (yellow dots). **E** The mesial surface features the parahippocampal gyrus (blue), dentate gyrus (green arrow), fimbria of the fornix (red arrow), and uncus (red dot). **F** Hippocampus anatomy includes the head (purple), body (green), tail (red), with fimbria and fornix (blue rectangles)

**Fig. 2:**
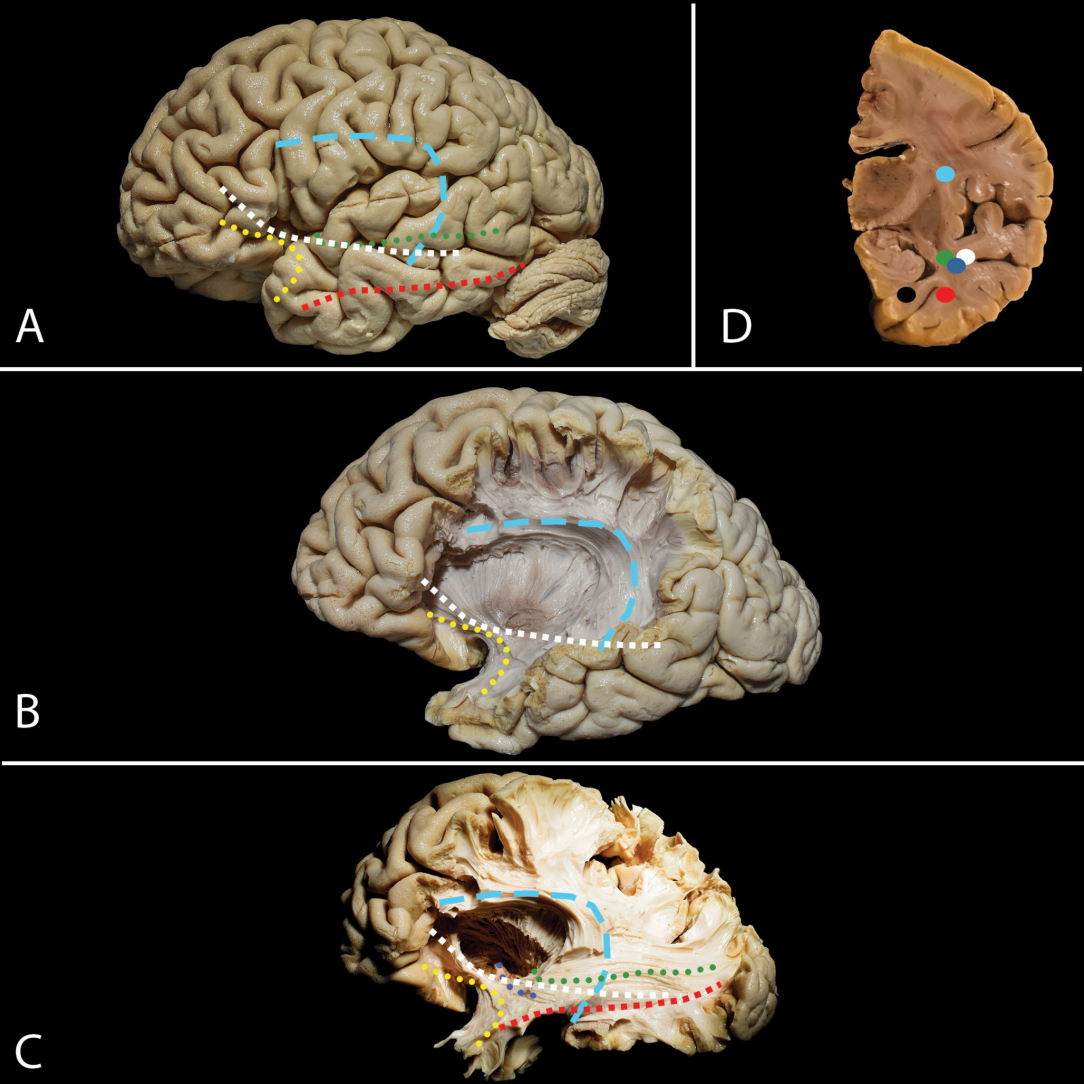
White Matter Tracts of the Temporal Lobe in Selective Amygdalohippocampectomy. **A**-**C**: **A** Lateral view of the brain hemisphere. **B** Superficial tracts near the temporal lobe. **C** Deep tracts related to the temporal lobe, including the arcuate/superior longitudinal fascicle complex (AF/SLF) (light blue rectangles), the uncinate fascicle (UF) (yellow dots), the inferior fronto-occipital fascicle (IFOF) (white squares), the optic radiation (green dots), the anterior commissure (blue dots), and the inferior longitudinal fasciculus (ILF) (red squares). **D** Coronal cut at the level of the temporal horn showing the optic radiation (green dot), IFOF (white dot), ILF (red dot), cingulum fibers (black dot), anterior commissure (blue dot) and AF/SLF (light blue dot)

**Fig. 3:**
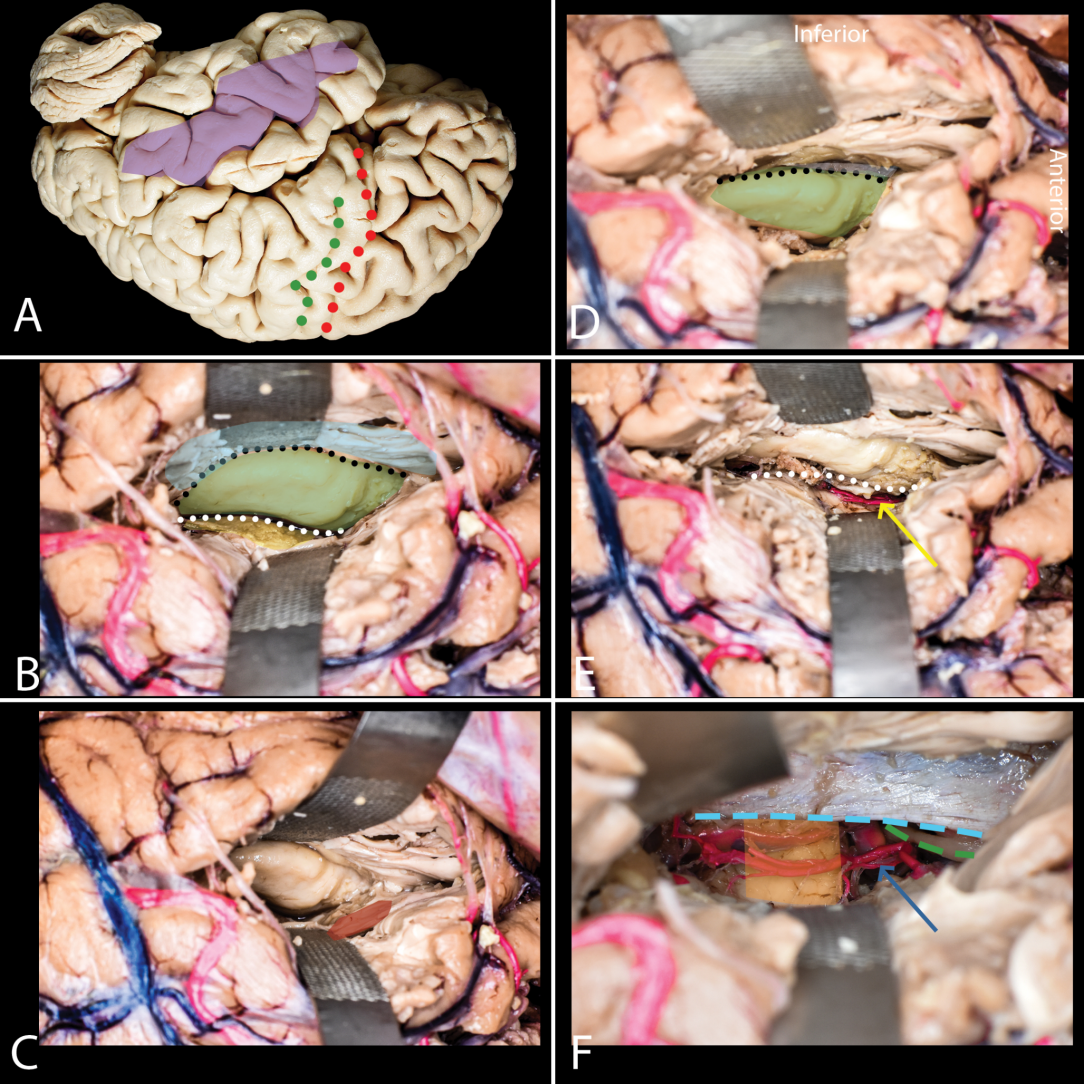
Trans-middle temporal gyrus approach (Transcortical approach). **A**-**F**: **A** Corticotomy in the middle temporal gyrus (purple) is performed based on hemisphere dominance. In the dominant hemisphere, it is anterior to the precentral sulcus (red), while in the non-dominant hemisphere, it is at the level of the central sulcus (green). **B** Opening the ventricle reveals the choroid plexus (yellow), choroidal fissure (white dots), hippocampus (green), lateral ventricle sulcus (black dots), and collateral eminence (blue). **C** The amygdala (red) is identified at the anterior temporal horn, medial and anterior to the hippocampus. **D** Subpial dissection of the parahippocampal gyrus is performed along the lateral ventricle sulcus (black dots), beneath the hippocampus proper (green). **E** The hippocampus is laterally displaced, exposing the choroidal fissure (white dots) and anterior choroidal artery (yellow arrow). **F** Final exposure of the tentorial incisura (light blue rectangle) after hippocampal resection, showing the cerebral peduncle (orange), basilar artery and its branches (blue arrow) and oculomotor nerve (green)

**Fig. 4:**
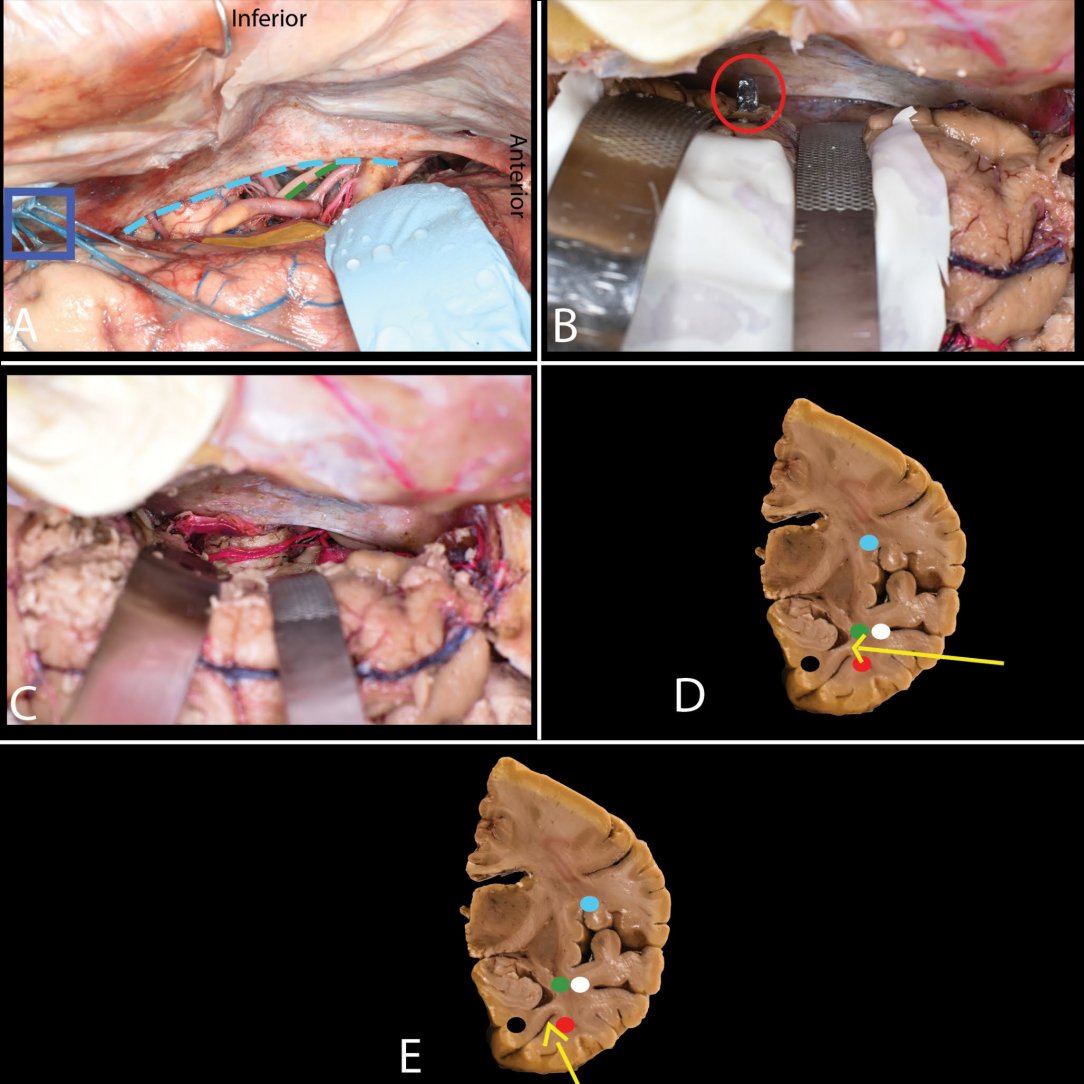
Subtemporal approach. **A**-**E**. **A** A retractor is placed under the brain, identifying the collateral sulcus. A cortical incision is made near the uncus (yellow), and care is taken to preserve the inferior anastomotic vein (blue square) and the oculomotor nerve (green rectangle), which is medial to the tentorial incisura (light blue rectangle). **B** A dissector (red circle) is placed from above within the lateral ventricular sulcus to present the projection of the temporal horn on the basal surface. **C** En bloc removal of the hippocampal body, similar to the trans-middle temporal approach, reveals structures of the tentorial incisura. **D** The yellow arrow shows the trajectory using the trans-middle temporal gyrus approach. This approach follows the trajectory of fibers forming the sagittal stratum, including the IFOF (white dot), anterior commissure, and optic radiation (green dot), while staying above the ILF (red dot). **E** The subtemporal approach preserves the IFOF and optic radiation but may interrupt ILF fibers

**Fig. 5:**
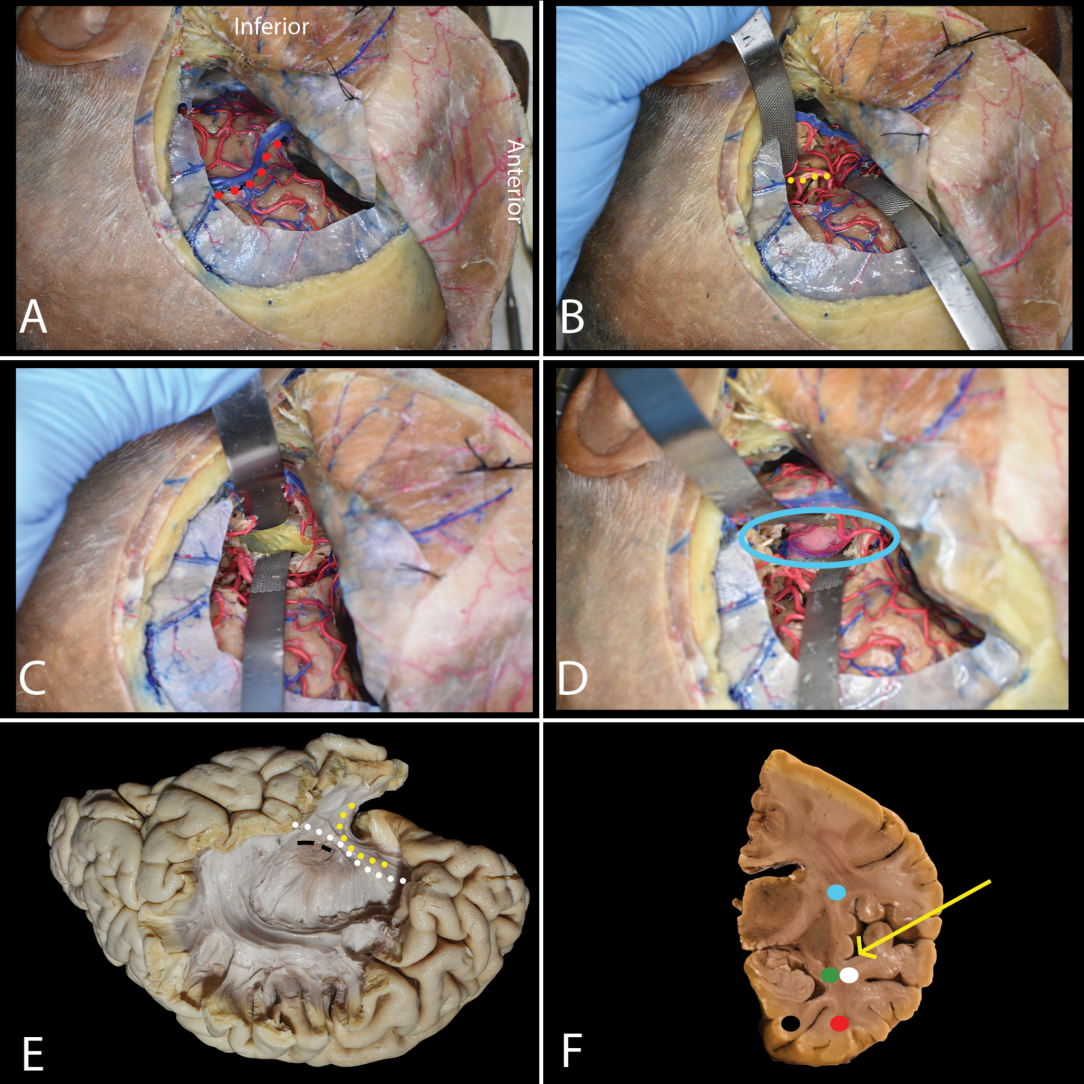
Transsylvian approach. **A**-**E**. **A** A pterional craniotomy is performed. The sylvian fissure (red dots) is opened from the internal carotid artery bifurcation to 2 cm beyond the MCA bifurcation. **B** The inferior temporal trunk (yellow dots) of the M2 segment of the MCA is identified and mobilized near the limen insulae. **C** Corticotomy within the inferior limiting sulcus reveals the amygdala and exposes the temporal horn (yellow). **D** The anterior choroidal artery (light blue circle) is preserved, and its branches to the hippocampus are coagulated. Care is taken to avoid injury to the cerebral peduncle (pink). **E** The approach passes through the limen insulae (black rectangle), risking interruption of the UF (yellow dots), IFOF (white dots), and anterior commissure. **F** The yellow arrow shows the trajectory using the thanssylvian approach

**Fig. 6:**
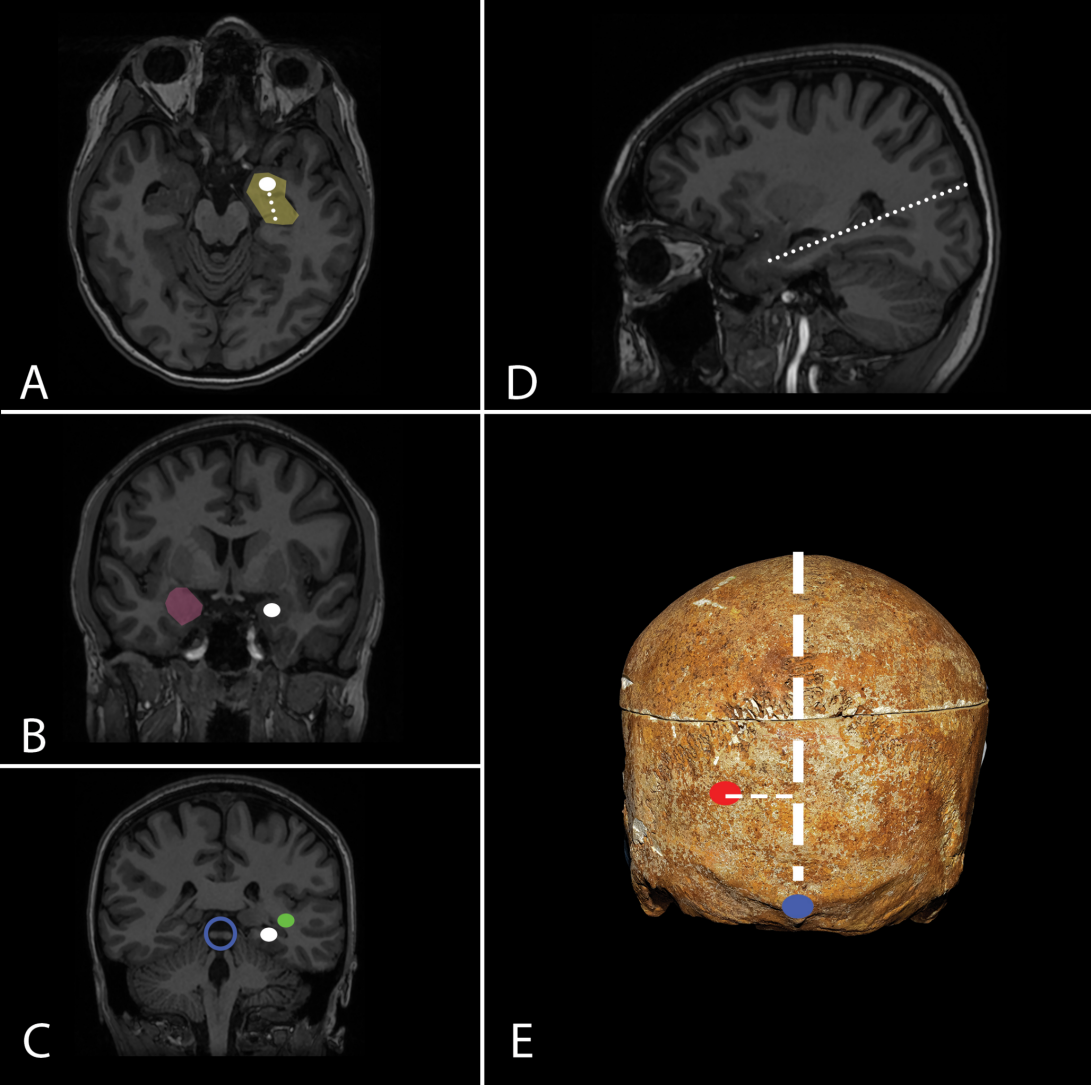
Laser interstitial thermal therapy (LITT). **A**-**E**. **A** A catheter with an optical fiber is inserted through a small bone opening, targeting the amygdala and hippocampus (yellow). **B** The catheter’s trajectory passes through the center of the amygdala (white dot), with the contralateral amygdala marked in pink. **C** At least 2.5 cm of the hippocampus is ablated, from the uncal apex posteriorly to the level of the lateral mesencephalic sulcus/quadrigeminal plate (blue circle), optic radiation (green dot) is located lateral to the temporal horn. **D** A steep trajectory (white dots) risks incomplete ablation and visual deficits. **E** The entry point (red dot) is typically 4–6 cm lateral to the midline (white rectangle) and 4–6 cm above the inion (blue dot), adjusted for individual anatomy to avoid critical structures

Mesial temporal lobe epilepsy (TLE) is the most common form of focal epilepsy among adult patients, with approximately one-third of them developing resistance to antiseizure medications [28]. Hippocampal sclerosis (HS) is the predominant neuropathological finding in these patients [22]. Within the mesial temporal lobe, other functional units such as the amygdala, uncus, dentate gyrus, parahippocampal gyrus, entorhinal cortex, and piriform cortex play crucial roles in the genesis of temporal seizures (Fig. 1). In cases of intractable TLE surgical treatment is considered the preferred management [29]. The primary objective of TLE surgery is to resect structures contributing to seizures genesis. The anterior temporal lobectomy (ATL) remains the gold standard technique in operative treatment of drug-resistant TLE. However, functional complexity makes mesiobasal temporal lobe an important structure and extensive resection through ATL often leads to substantial adverse effects, mostly associated with cognitive and visual impairment. To reduce the extent of the resection and improve neuropsychological outcomes, more selective methods – such as selective amygdalohippocampectomy (SAHE) have ben developed. Some comparative studies of standard ATL and SAHE indicate that similar seizure freedom outcomes can be expected [25, 32]. Notably, SAHE demonstrates superiority in terms of neuropsychological outcomes, attributed to the preservation of cortical and subcortical structures within the temporal pole (Fig. 2). The concept of selective amygdalohippocampectomy originated from the technique initially described by Niemeyer in the 1950s [24]. Three primary approaches—transsylvian (tsSAHE), transcortical (tcSAHE), and subtemporal (stSAHE) — are commonly employed. Additionally, minimally invasive techniques such as laser interstitial thermal therapy (LITT) have gained popularity among neurosurgeons due to their comparable outcomes to SAHE and lower risk of adverse effects, including cognitive impact [18]. These approaches differ in technical complexity, given the anatomical variations encountered along each surgical trajectory. The choice of a specific approach is mainly based on the surgeon’s preference and experience with the respective technique [13, 24, 34]. The objective of this study is to provide anatomical and surgical details of tcSAHE (Fig. 3), stSAHE (Fig. 4), tsSAHE (Fig. 5), or LITT (Fig. 6) technique to shed more light on the critical anatomical landmarks and technical nuances associated witch each method to aid surgeons with intraoperative decision-making and selection of the most appropriate trajectory to minimize the risk of complications.

## Materials and methods

Five fresh human head and brain specimens (10 sides) were dissected at the Surgical Neuroanatomy Laboratory of the Department of Neurological Surgery at the Medical University of Warsaw. The common carotid arteries, vertebral arteries, and internal jugular veins were isolated, cannulated, and injected with red and blue silicone for enhanced visualization. To facilitate white matter dissection, the adult cerebral hemispheres were prepared using Klingler’s preparation technique [8]. The cortical surface was assessed using the naked eye. Next, white matter dissection was performed using microscopic magnification and microsurgical tools to expose the white matter tracts and ventricular system within the temporal lobe. A digital camera (NikonD7200 with a Nikon DX 35 mm lens; 1:1.8 G) was used for image documentation. The study was approved by the Bioethics Committee of Medical University of Warsaw, approval number AKBE/190/2023.

## Results

### Anatomy of the Temporal Lobe (Fig. 1)

On the convexity of the cranium, the temporal lobe approximately corresponds to the squamous part of the temporal bone. After craniotomy and dural opening, the lateral surface of the temporal lobe with three parallel gyri becomes exposed. The temporal horn and mesial temporal structures are not visible from a lateral perspective, located deep to the middle temporal gyrus. When splitting the sylvian fissure, the dissection plane follows the superior surface of the lobe, which continues with the insula at the level of the inferior limiting sulcus. The most consistent anatomical landmark on the basal surface of the hemisphere is the collateral sulcus, which is identified laterally to the parahippocampal gyrus. The uncus is the anterior part of the parahippocampal gyrus, and the rhinal sulcus marks their lateral boundary. These two structures together form the mesial structures of the temporal lobe. Lateral to the collateral sulcus, the fusiform gyrus is identified, which is laterally separated from the inferior temporal gyrus by the lateral occipitotemporal sulcus. The mesial temporal lobe is divided into three segments: anterior, medial, and posterior. The anterior segment starts at the anterior end of the rhinal sulcus and ends at the inferior choroidal point. The medial segment lies at the level of the quadrigeminal plate, and the posterior segment ends at the level of the line connecting the preoccipital notch with the parieto-occipital sulcus. The temporal horn extends from the atrium to the end of the lateral ventricle within the temporal lobe, which is located approximately 2.5 cm from the temporal pole. The base of the ventricle is formed medially by the hippocampus and laterally by the collateral eminence, which corresponds to the collateral sulcus on the outer surface. The only structure on the medial wall is the choroid fissure, which is marked superiorly by the thalamus and inferiorly by the fimbria of the fornix. Within the ventricle, the amygdala and the head of the hippocampus are separated by the uncal recess, which corresponds to the uncal apex on the mesial surface. On the mesial surface above the parahippocampal gyrus and the hippocampal sulcus, the dentate gyrus and fimbria of the fornix are identified. Within the uncus the anterior segment is formed by the amygdala, while the upper part of the posterior segment is formed by the anterior part of the head of the hippocampus. The hippocampus is a structure about 5 cm long on the mesial surface of the temporal horn, located on the parahippocampal gyrus. It consists of three parts: the head, body, and tail. Just behind the head, the initial segment of the fimbria and the choroid plexus within the inferior choroidal point are identified. Regardless of the approach, the resection of the hippocampus involves anterior disconnection (uncal recess), lateral disconnection (entering the parahippocampal gyrus), posterior disconnection (hippocampus at the level of the quadrigeminal plate), and medial disconnection (opening of the choroidal fissure).

### Anatomy of the White Matter Tracts - In Focus of Selective Amygdalohippocampectomy (Fig. 2)

From a lateral perspective, the most superficial tract encountered during white matter dissection in the temporal region is the AF/SLF complex. This complex is found within the posterior portions of the superior and middle gyri. Anteriorly within the temporal lobe, the UF is identified, which, at the level of the limen insulae, runs parallel and ventral to the IFOF. Along the anterior-posterior trajectory, the fibers of the IFOF merge with the OR, anterior commissure (blue dots), and inferior longitudinal fasciculus (ILF), thus forming the sagittal stratum and the lateral wall of the ventricle. The OR and IFOF constitute the superior/lateral wall of the temporal horn and do not extend beyond the inferior temporal sulcus. Approaching the mesial structures from an inferior trajectory, we can identify the ILF, with most of its fibres situated inferior to the temporal horn of the lateral ventricle. Furthermore, the cingulum fibers within the parahippocampal gyrus are interrupted before reaching the ventricle.

### Trans-middle temporal gyrus approach (Transcortical approach)(Fig. 3)

After placing the patient in the lateral or supine position, with the long axis of the head parallel to the floor, a linear skin incision above the zygoma is performed. Following soft tissue dissection and splitting of the temporalis muscle along its fibers, cranial anatomical landmarks must be visualized. The squamous suture corresponds to the lateral sulcus, and its highest point corresponds to the Inferior Rolandic Point (IRP). This point is located approximately 4 cm above the preauricular depression, which is identified anteriorly to the tragus. After dural incision and brain exposure, a 2 to 3 cm corticotomy is performed over the middle temporal gyrus. The placement of the corticotomy is determined based on hemisphere dominance in terms of language function. In the dominant hemisphere, the corticotomy is placed within the middle temporal gyrus anterior to the precentral sulcus. This places the cortical incision within the limits of the standard temporal resection in the dominant hemisphere, which does not result in speech disturbances during long-term follow-up. In the non-dominant hemisphere, it is placed at the level of the central sulcus, which is marked by the highest point of the squamous suture. The height of the corticotomy is approximately 4–5 mm, allowing for proper lateral exposure of the temporal horn of the ventricle. After opening the ventricle, identification of key anatomical landmarks is performed. The first landmark to identify is the choroid plexus, which corresponds to the choroidal fissure, located medially to the hippocampus. Following the intraventricular surface of the hippocampus laterally, the lateral ventricle sulcus is identified. This sulcus marks the border between the hippocampus and collateral eminence and serves as the entry point to the parahippocampal gyrus. The amygdala can be identified at the most anterior point of the temporal horn, located anterior and medial to the hippocampus on the medial and inferior wall of the ventricle. After the identification of key anatomical landmarks, a subpial dissection of the parahippocampal gyrus, which is located under the hippocampus proper, is performed along the lateral ventricle sulcus in an anterior-posterior direction, until the edge of the tentorium, which is visualized through the pia. The anterior end of the resection is carried out until the uncus is removed, while the posterior border of the resection is marked by the lateral mesencephalic sulcus. Resecting the parahippocampal gyrus allows for the lateral and inferior lateralization of the hippocampus in this free space, which enables the opening of the choroidal fissure and exposure of the fimbria of the fornix and visualization of the anterior choroidal artery and its branches under the pia. In each step, care should be taken to consider the posterior cerebral artery (PCA) and anterior choroidal artery when dissecting under the pia. Before the removal of the hippocampal complex, care should be taken to preserve arteries arising from the anterior and posterior choroidal arteries. The residual parahippocampal gyrus and amygdala are resected within the borders of the medial and inferior pial margins. Special attention is given to avoid the cerebral peduncle and the oculomotor nerve, which are identified under the pia on the mesial surface of the resection, medial to the tentorial incisura. The most dorso-medial extent of the amygdala, corresponding to the optic tract, should also be identified and taken into consideration (Table 1).

**Table 1:** Summary of white matter tracts and vascular structures at risk with each approach

Approach	White matter tracts at risk of injury	Anatomical structures at risk of injury
Trancortical approach	IFOF, AC, OR	-
Subtemporal approach	ILF	Inferior anastomotic vein
Transsylvian approach	IFOF, UF	Middle cerebral artery
LITT	OR	-
Anterior temporal lobe resection	AF/SLF complex UF, IFOF, OR, AC, ILF	Middle cerebral artery

### Subtemporal approach (Fig. 4)

The patient is placed in the supine position with a roll under the shoulder or in the lateral position with the long axis of the head rotated. A skin incision is made around the pinna of the ear, exposing the zygoma, and soft tissue dissection is performed up to the level of the external acoustic meatus. A subtemporal craniotomy is then performed, removing the squamous part of the temporal bone as low as possible using a drill. The dura is reflected towards the skull base, and the inferior and middle temporal gyri are exposed. A brain retractor is placed about 2 cm on the undersurface of the brain, and the collateral sulcus is identified. The cortical incision, which is made at a midpoint of the uncus, can be identified about 1.0–1.5 cm posterior to the point at which the oculomotor nerve enters the tentorial edge. When retracting the temporal lobe, care is taken to preserve the inferior anastomotic vein (also called the vein of Labbe). The individual anatomy of the vein is the main limitation of the approach in terms of working space. Additional brain relaxation before the main part of the surgery can be achieved by opening the basal cisterns and releasing cerebrospinal fluid. A corticotomy is performed within the fusiform gyrus when there is a lack of anatomical landmarks due to being covered by the arachnoid; neuronavigation can be applied, and the temporal horn is entered at a depth of approximately 1.5 cm. This exposes the amygdala, uncal recess, head and body of the hippocampus, and the inferior choroidal point – the perspective of the ventricle is like the trans-middle temporal gyrus approach. The steps for en bloc removal of the hippocampal body are similar to the trans-middle temporal gyrus approach, and the final outcome of resection reveals the structures of the tentorial incisura.

### Transsylvian approach

After placing the patient in a supine position with the torso elevated, the head is rotated approximately 30 degrees to the non-operated side, and the vertex is tilted down, with the malar eminence as the most superior point. A standard pterional craniotomy over the sylvian fissure, identified by the groove between the frontal and temporal bones, is performed. Once the bone flap is elevated, a drill is used to flatten the sphenoid bone to the level of the superior orbital fissure, which is identified by the orbitomeningeal trunk, to facilitate exposure. The dura is opened in a semicircular fashion and reflected over the sphenoid ridge. The sylvian fissure is then opened from the area of the internal carotid artery bifurcation to approximately 2 cm distal to the middle cerebral artery (MCA) bifurcation or about 2 cm behind the tip of the pars triangularis on the surface. The inferior (temporal) trunk of the M2 segment of the MCA is identified as it runs along the inferior part of the insular sulcus, close to the limen insulae, and usually needs to be mobilized before corticotomy. The corticotomy is performed within the inferior limiting sulcus of the insula, and a few millimeters beneath the cortical surface, the amygdala is located. The amygdala is removed, and the entire temporal horn is exposed and opened until the ventricular trigone – a confluence of temporal and occipital horns of the lateral ventricle is identified. After the removal of the amygdala, the anterior part of the parahippocampal gyrus is subpially resected, and structures within the ambient and interpeduncular cisterns are identified. Attention should be paid to the anterior choroidal artery, which can be found in connection with the anterior part of the parahippocampal gyrus and uncus. The main trunk of the artery and medial branches must be preserved, while the lateral branches (often referred to as the uncus artery) should be identified, coagulated, and divided. Posteriorly, attention should be given to the choroidal arteries originating from the posterior cerebral artery. Care should be taken if there is a mesial herniation of the structures, as it carries a risk of injury to the structures within the tentorial incisura, including the cerebral peduncle, which is why subpial dissection is recommended. With all these disconnections, the hippocampus is removed in one piece, along with some of the parahippocampal gyrus. The chosen approach involves passing through the limen insulae, which poses a risk of interrupting the UF, IFOF, and anterior commissure.

### Laser interstitial thermal therapy (LITT)

MR-guided amygdalohippocampectomy using laser interstitial thermal therapy (LITT) is an approach in the management of temporal lobe epilepsy (TLE), which allows for the precise delivery of a focused dose of thermal energy and selective ablation of targeted tissue. The patient is placed in the supine semisitting position with the head flexed to properly expose the parieto-occipital region. A short linear skin incision is made, and a limited bone opening is performed through which a catheter containing an optical fiber is inserted. The target point on the axial plane is placed within the amygdala and hippocampus. With the planned linear trajectory, the catheter has to traverse the head and most of the body of the hippocampus, as well as the inferior part of the amygdala. To achieve that, the target point is placed on the amygdala and must go through the concavity of the hippocampus. On a coronal plane, it is necessary to adjust the trajectory to pass through the center of the amygdala, with the amygdala on the contralateral side marked in pink. The general objective is to ablate at least 2.5 cm of the length of the hippocampus from the uncal apex posteriorly to the level of the lateral mesencephalic sulcus/quadrigeminal plate. In the axial plane, to achieve complete ablation of the hippocampus and avoid unnecessary trauma, it is essential to stay within the center of the hippocampal head. A location that is too lateral may result in incomplete ablation of mesial structures, while a location that is too medial can lead to oculomotor and trochlear cranial nerve injury within the tentorial incisura. In the sagittal plane, a trajectory that is too steep may result in incomplete ablation of the uncal apex and amygdala, as well as damage to the lateral geniculate nucleus, resulting in visual field deficits. Overall, to create the desired ablation volume, 3–5 series of ablations are performed as the optical fiber is being withdrawn, with progressively lower intensity of applied thermal energy, to avoid thalamus and OR injury while moving posteriorly. Usually, the entry point is located somewhere between 4 and 6 cm lateral to the midline and 4–6 cm above the inion. The variations in the localization of the entry point are related to the individual anatomy, as the trajectory must avoid the ventricle, choroid plexus, and vascular structures.

## Discussion

At present, ATL and SAHE are two primary, safe and effective techniques in intractable temporal lobe epilepsy management. Literature review indicates low complication rates (ranging from 2.9 to 8.4%) with common surgical complications including cerebrospinal fluid leak, aseptic meningitis, bacterial infections, intracranial hematomas and neurological complications, such as visual field deficits and speech impairment – particularly transient aphasia, especially following the transcortical approach [1]. In terms of seizure control, while many studies report that SAHE and LITT have comparable outcomes to ATL, some suggest that selective procedures may be inferior in achieving seizure freedom [4, 5, 17, 19, 33]. An ATL involves a more extensive resection and, therefore, may provide a higher likelihood of completely removing structures associated with seizure generation. However, selective techniques are increasingly gaining popularity due to its suspected efficacy in minimizing the risk of cognitive deficits [23, 31]. Understanding the intricate anatomy of white matter tracts in the temporal lobe and surrounding structures is critical in planning this procedure and minimizing postoperative complications, especially in terms of neuropsychological outcome [15].

The most superficial white matter tract encountered in temporal lobe dissections is the AF/SLF complex, which plays a crucial role in language and speech processing [16, 30]. The nomenclature used to describe these complex and overlapping white matter tracts that terminates within the posterior parts of the superior and middle temporal gyri in the region related to Wernicke’s area remains a subject of debate [3]. This emphasizes the importance of preserving these fibers, especially in the dominant hemisphere. Injury to the AF/SLF complex during surgical approaches could result in significant language deficits [16, 30]. Among the limited approaches, direct injury to these regions, often referred to as Wernicke’s area, is mainly associated with the transcortical approach through the middle temporal gyrus. Proper placement of the cortical incision in relation to Wernicke’s area is crucial and can be guided by intraoperative neuromonitoring during awake procedures or by preoperative functional MRI [6].

Anteriorly, the UF and the IFOF are encountered. The IFOF forms the sagittal stratum, which consists of key connections between the temporal, frontal, and occipital regions [9, 20]. The UF connects the anterior temporal lobe with the orbitofrontal cortex and plays a role in memory and emotional processing [26]. The IFOF runs parallel and ventral to the UF, merging with the OR, anterior commissure, and ILF along its course [2, 21]. Since these tracts are essential for maintaining visual and cognitive functions, damage to the IFOF and OR during temporal lobe surgery can result in visual field deficits and cognitive impairments [2, 21]. These tracts are mainly at risk during the transsylvian approach, which involves transection of the limen insulae, where both tracts run in close proximity to each other [7]. Using the middle temporal gyrus approach, the trajectory runs parallel to the fibres that eventually form the sagittal stratum, including the IFOF, anterior commissure, OR, and above the ILF. With subtemporal approach all the tracts located laterally to the ventricle (IFOF; OR) are preserved. However, the trajectory of this approach may result in the interruption of ILF fibres.

Approaching the mesial temporal structures from below requires caution due to the proximity of white matter tracts such as the ILF and cingulum fibers. The ILF, which predominantly carries fibers between the occipital and temporal lobes, plays an integral role in visual processing and object recognition [11]. Disruption of the ILF during surgical resection may lead to impairments in visual perception, memory, and processing of visual stimuli, especially with the subtemporal approach. From a technical standpoint, the LITT technique offers a minimally invasive approach that allows for the sparing of these important tracts, as it precisely targets the amygdala and hippocampus while avoiding damage to surrounding structures [18]. Despite consideration of key white matter tracts, the challenge in selecting an approach includes the proximity of the parahippocampal gyrus and hippocampus to critical structures like the middle cerebral artery, anterior choroidal artery, the fimbria of the fornix, and the brain stem. Damage to these structures during SAHE could result in deficits in memory, visual processing, spatial navigation, or severe neurological motor deficits.

At the moment, there remains insufficient evidence to definitively determine which of the approaches discussed in this study yields superior neuropsychological outcomes, highlighting the need for further research in this area. However, of note is the fact that even with a detailed understanding of white matter tract anatomy and successful avoidance of vascular injury, sometimes SAHE may result in poorer neuropsychological outcomes compared to ATL [14,27]. The reasons for this difference are still debated in the literature. Possible explanation is the suspected lower rate of long-term seizure freedom with SAHE, as persistent seizures may contribute to adverse cognitive and neuropsychological outcomes. Consequently, potential improvements in neuropsychological performance following SAHE may be offset by cognitive decline associated with ongoing seizure activity, in contrast to the outcomes observed with ATL [10, 12].

A limitation of this study worth highlighting is the small amount of examined specimens (five human heads). Small sample size may reduce the generalizability of the findings, particularly given the complexity of temporal lobe anatomy and variability in the courses of white matter tracts. Further studies with larger sample sizes are needed to confirm and expand upon these anatomical observations.

## Conclusion

Overall, the preservation of white matter tracts in the temporal lobe is critical for the successful neuropsychological outcome of selective amygdalohippocampectomy. Understanding the anatomical course of the AF/SLF complex, UF, IFOF, ILF, and OR helps in minimizing postoperative cognitive, visual, and language deficits. Surgeons must carefully tailor their approach to the individual anatomy of the patient, utilizing modern neuronavigation and intraoperative monitoring to achieve maximal lesion resection while preserving vital structures. Further research and refinement of surgical techniques will continue to reduce the risks of white matter tract damage, enhancing both seizure control and the preservation of cognitive functions in TLE patients.

## Data Availability

No datasets were generated or analysed during the current study.
